# First Prenatal Diagnosis of a Niemann–Pick Disease Type C2 Revealed by a Cystic Hygroma: A Case Report and Review of the Literature

**DOI:** 10.3389/fendo.2018.00292

**Published:** 2018-06-06

**Authors:** Liana Ples, Romina-Marina Sima, Florina Nedelea, Marius Moga

**Affiliations:** ^1^UMF, Carol Davila, Bucharest, Romania; ^2^St. John Hospital, Bucur Maternity, Bucharest, Romania; ^3^Filantropia Clinical Hospital, Bucharest, Romania; ^4^Faculty of Medicine, Transilvania University of Brasov, Brasov, Romania

**Keywords:** cystic hygroma, unusual association, Niemann–Pick disease, prenatal diagnosis, homozygous mutation

## Abstract

**Background:**

The importance of fetal nuchal translucency was highlighted in the early 1990s as a useful first-trimester marker to identify fetal chromosomal abnormalities. Here, we report the prenatal diagnosis of a fetus with Niemann–Pick disease type C initially identified by first-trimester ultrasonographic markers and eventually confirmed by extensive genetic evaluation.

**Case presentation:**

The fetus of a 30-year-old woman exhibited a cystic hygroma in the first trimester of pregnancy. The woman underwent chorionic villus sampling with extensive genetic investigations to identify the genetic cause of the ultrasonographic findings. Owing to normal karyotype results, further evaluation of 1,024 genes underlying structural abnormalities was performed. This test identified a homozygous mutation of the *NPC2* gene (OMIM 601015), which has been reported to be pathogenic and responsible for Niemann–Pick disease type C2 (NPD-C2). Genetic evaluation of the parents found them to be carriers. Considering the poor prognosis, the parents decided to terminate the pregnancy. Ultrasonographic screening during the subsequent pregnancy showed normal findings; however, molecular testing for the previous familial mutation c.441 + 1G>A identified the fetus as homozygous for this mutation. Therefore, the parent chose to terminate the subsequent pregnancy as well.

**Conclusion:**

We report the first prenatal diagnosis of NPD-C2 based on a cystic hygroma found during the first trimester of pregnancy as the sole indicator.

## Introduction

In the early 1990s, Nicolaides et al. revealed the importance of fetal nuchal translucency (NT) as a first-trimester indicator of fetal chromosomal abnormalities (1). Since then, more than 2,000 articles have been published describing NT as a first-trimester prenatal diagnostic indicator of different chromosomal or monogenic diseases, including metabolic disorders. Herein, we report the prenatal diagnosis of Niemann–Pick disease type C2 (NPD-C2), initially identified by this first-trimester ultrasonographic anomaly and subsequently confirmed by extensive genetic evaluation.

## Case Presentation

**Figure 1:**
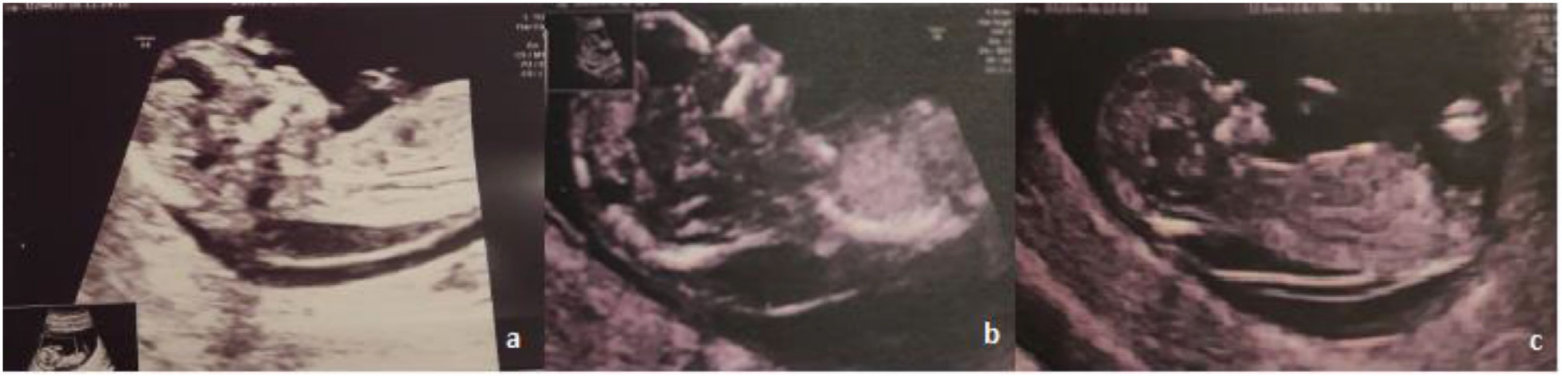
Ultrasonographic images of the fetus.

A 30-year-old woman was referred to her obstetrician for pregnancy monitoring. The first ultrasonographic examination confirmed a 7-week pregnancy. She had delivered one child after a previous normal pregnancy and had no history of abortion. Routine biochemical tests yielded normal red blood cell count, hemoglobin level, renal and hepatic function, and blood sugar level values for a woman in the first trimester of pregnancy. First-trimester ultrasonographic screening for chromosomal anomalies revealed a large NT of 6.4–7 mm, with thin septae limited to the back of the fetus; the nasal bone was present, and normal blood flow was observed through the ductus venosus and tricuspid valve (Figure 1).

The management of the case and challenges presented are detailed in Table 1.

**Table 1:** The diagnostic steps and challenges.

Diagnostic step	Challenges
Fetal thorough morphologic assessment at the time of first diagnosis	Small fetus (11 + 3 weeks), absence of other aneuploidy signs

Chorionic villous sample and fetal karyotype assessment	Normal euploidic fetus XX

Ultrasonographic evaluation of the fetal anatomy at 14 weeks double-checked with another fetal specialist	Normal fetal heart (in absence of chromosomal anomalies, chronic heart disease is the most frequent cause for an increased NT) Normal fetal anatomy [absence of trigger signs that would prompt a search for syndromes (Noonan, Fryns) or other structural anomalies (diaphragmatic hernia, musculoskeletal anomalies, urinary malformations, fetal tumors, and CCAM) that can cause increased NT]

*NT, nuchal translucency; CCAM, congenital cystic adenomatoid malformation*.

The combined risk for trisomies 21, 13, and 18 was 1/560, but considering the known association between these heterogeneous genetic syndromes and cystic hygroma, prenatal screening was recommended.

**Figure 2:**
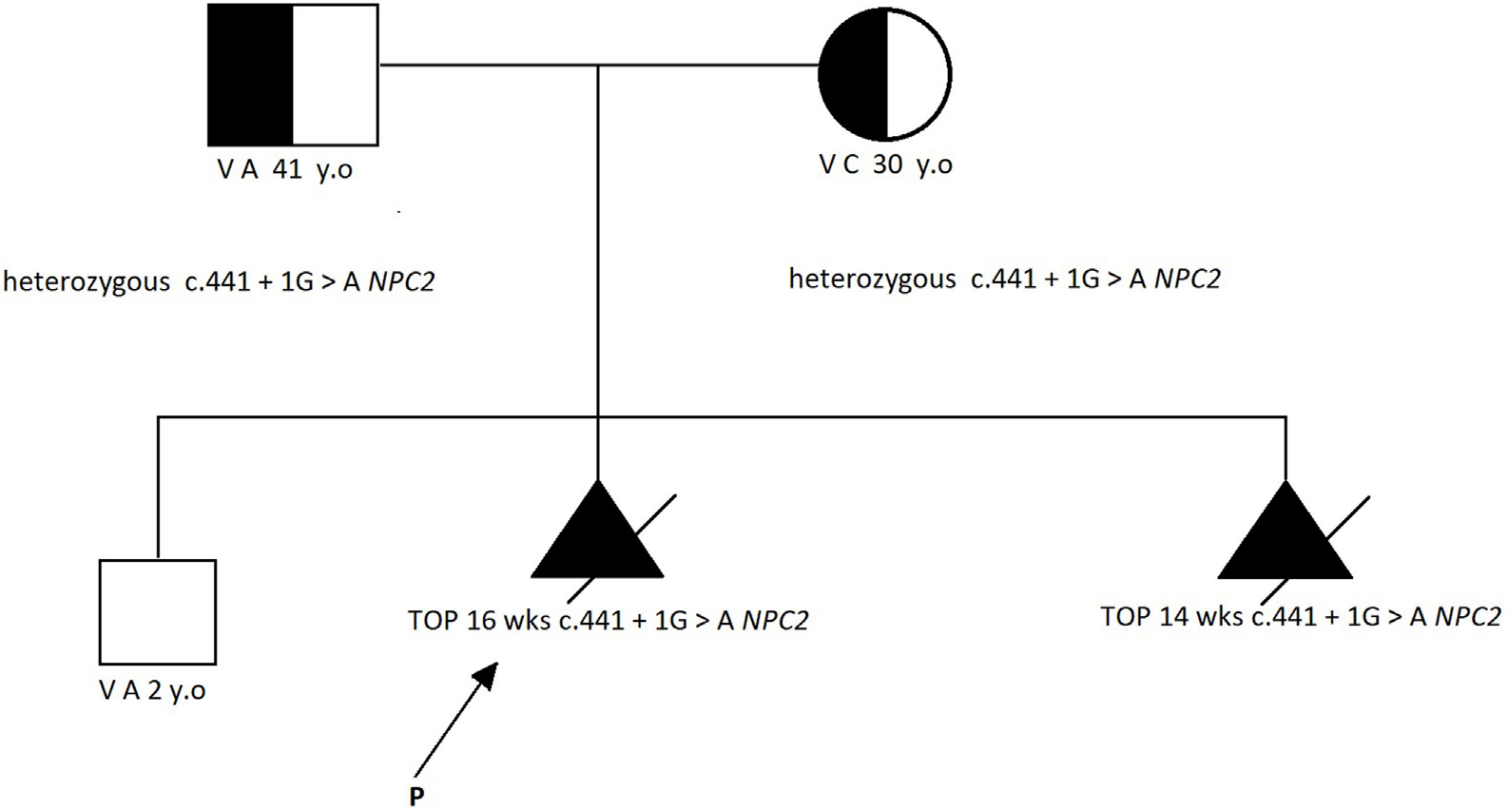
The family pedigree [according to Ref. (2)].

Karyotype analysis and DNA extraction from chorionic villi were performed. Owing to the normal results of the karyotype and multiple genetic syndromes associated with increased NT, we pursued next-generation sequencing of 1,024 genes that have been associated with several structural abnormalities; this identified the known homozygous pathogenic mutation c.441 + 1G>A of the *NPC2* gene (NM_006432.3, OMIM 601015). The mutation has been reported in international databases as pathogenic (3) and responsible for NPD-C2. Genetic testing of the parents and the previous offspring revealed that they were both heterozygous (carriers) for the abovementioned mutation, although their first child was not affected (Figure 2).

Considering the poor prognosis, the parents decided to terminate the pregnancy at 16 weeks, which was uneventful.

Six months later, the patient presented with a new pregnancy at a gestational age of 11 + 3 weeks. Ultrasonographic screening showed normal findings, with an NT of 6 mm. Chorionic villus sampling was performed due to the high recurrence risk of Niemann–Pick disease. The karyotype was found to be normal. However, molecular testing revealed that the fetus was homozygous for the previously identified familial mutation c.441 + 1G>A; hence, the pregnancy was terminated at 14 weeks.

## Discussion

Niemann reported a child with hepatosplenomegaly and rapid neurodegeneration who died in early childhood (4). He described this illness as different from the storage disease described by Philippe Gaucher in 1882. Later, Pick defined the pathology of the disease, in which foam cells infiltrated peripheral tissues (5). Klenk discovered that tissue levels of sphingomyelin are elevated in this disorder (6). Since then, three different types of the disease, Niemann–Pick disease type A (NPD-A), Niemann–Pick disease type B (NPD-B), and Niemann–Pick disease type C (NPD-C), have been identified. Roscoe Brady discovered acid sphingomyelinase deficiency in 1966 and classified Niemann–Pick diseases into types A and B according to biochemistry; however, the enzymatic deficiency associated with type C disease remained uncertain (7).

Niemann–Pick disease type A was described as the acute neuronopathic form. NPD-B is pan-ethnic, with a later onset than NPD-A as well as less severity. The overall prevalence of types A and B combined is estimated to be 1:250,000, although NPD-A is highest among Ashkenazi Jews (approximately 1 per 70–100 persons) (8). The American College of Medical Genetics and Genomics recommends routine carrier screening for the following nine disorders in this population: Tay–Sachs disease, Canavan disease, cystic fibrosis, familial dysautonomia, mucolipidosis IV, NPD-A, Fanconi anemia group C, Bloom syndrome, and Gaucher disease, owing to carrier detection rates as high as ≥90% and population carrier frequencies of ≥1% (9).

The American College of Obstetricians and Gynecologists Committee on Genetics also recommends that all Ashkenazi Jews undergo routine carrier screening for four of the most common genetic disorders that are either lethal or associated with significant morbidity: Tay–Sachs disease, Canavan disease, cystic fibrosis, and familial dysautonomia, and also suggests screening with an expanded panel that adds testing for Bloom syndrome, familial hyperinsulinemia, Fanconi anemia group C, Gaucher disease, glycogen storage disease type I, Joubert syndrome, Maple syrup urine disease, mucolipidosis IV, NPD-A, and Usher syndrome (10).

Generally, Niemann–Pick disease is initially suspected upon the observation of variable phenotypic manifestations, but the final diagnoses of NPD-A and NPD-B are based on the identification of acid sphingomyelinase deficiency. The definitive diagnosis of NPD-C requires the demonstration of abnormal intracellular cholesterol trafficking by the impaired response to low-density lipoprotein cholesterol loading in cultured fibroblasts (11).

Using the landmark criteria for identifying Niemann–Pick disease as described by Crocker and Farber in 1958 (12), Crocker first identified NPD-C in 1961 (13). NPD-C can be diagnosed from the perinatal period until late adulthood (14), and its estimated prevalence is approximately 1:100,000 in Europe. More than 85% of patients have systemic involvement of the liver, spleen, or lung before neurological symptoms manifest. For NPD-C, two genotypes related to mutations in the *NPC1* gene (NPD-C1) or the *NPC2* gene (NPD-C2) have been identified (15).

The existence of NPD-C as a distinct entity remained disputed until a decisive diagnosis was made in Roscoe Brady’s laboratory by Pentchev et al. (16). Pentchev et al. redefined the biochemical and pathological phenotypes of the NPC by discovering unusual oxysterols on the protein, which was critical to understanding the progress of this disease (17). Cultured fibroblasts and other tissues from individuals with NPD-C demonstrated highly variable levels of acid sphingomyelinase activity (18, 19). In terms of NPD-C pathogenesis, it was observed that the NPC1 and NPC2 proteins bind to cholesterol derived from endocytosed lipoproteins and transport it to the lysosomal membrane (20). Genetic defects in *NPC1* and *NPC2* are responsible for neurodegenerative disorders characterized by the accumulation of cholesterol and other lipids in lysosomes (21). Freeze-fracture electron microscopy demonstrated that the *NPC* yeast orthologs *Ncr1p* and *Npc2p* are vital for the formation and expansion of raft-like domains in the vacuolar membrane in the stationary and acute nitrogen starvation phases. Furthermore, the multivesicular body pathway has an important role in inducing microautophagy in response to acute nitrogen starvation. These data show that NPC proteins promote microautophagy in the stationary phase and under nitrogen starvation conditions, thereby increasing sterol levels in the limiting membrane of the vacuole (22).

Data from patients reported to date allow for the following conclusions to be derived: (1) the combination of clinical features found in patients with NPD-C is unique but not pathognomonic for juvenile dystonic lipidosis; (2) NPD-C can be initially identified by the examination of nerve bundle fibroblasts with electron microscopy, but definitive diagnosis requires determining the lipid profile from a liver or spleen biopsy in the absence of demonstrable enzymatic deficiency; (3) intralysosomal storage differs between the central nervous system and the spleen; and (4) juvenile dystonic lipidosis represents a juvenile variant of NPD-C (23).

Prenatal testing for pregnancies at risk of NPD-A or NPD-B includes measuring sphingomyelinase activity in amniotic fibroblasts (24, 25). Screening for population-specific mutations is feasible for individuals of Ashkenazi Jewish and North African descent (26); however, as mentioned earlier, there are two separate disease gene loci for NPD-C, the *NPC1* gene on chromosome 18q11-q12, and the *NPC2* gene (also called the *HE1* gene) on chromosome 14q24.3 (27–29).

Ultrasonographic examination of fetuses and placentas plays a major role in the diagnosis of metabolic disorders (30). The known metabolic disorders associated with non-immune hydrops are Gaucher disease, GM1 gangliosidosis type 1, Hurler syndrome, and mucolipidosis type I. In 1990, Meizner et al. reported an association of non-immune hydrops fetalis with Niemann–Pick disease for the first time. Ultrasonographic, biochemical, and histochemical studies of the placentas of four unrelated women carrying fetuses afflicted with NPD-A, who underwent prostaglandin-induced abortions at approximately the 19th week of gestation, demonstrated sphingomyelin accumulation. This indicates the essential role of the enzyme sphingomyelinase in the early stages of gestation (31).

In 2016, Colin et al. presented the third worldwide case of an *in utero* NPC diagnosis. Ultrasonographic screening at 22 weeks of gestation detected fetal ascites, and hepatomegaly and splenomegaly progressively appeared. There were no signs of placentomegaly or hydrops fetalis. The diagnosis of NPD-C was prenatally confirmed by the filipin test, *NPC1* sequencing, and a multiplex ligation-dependent probe amplification assay, which revealed a maternal missense mutation (c.2608T>C; p.Ser870Pro) and a paternal deletion of exons 5–25 (32). This prenatal case of NPC suggests that even in the absence of a family history of hydrops fetalis, Niemann–Pick disease should be considered a possible diagnosis. To the best of our knowledge, there have been no reported NPC diagnoses *in utero* during the first trimester of pregnancy.

The only study that aimed to assess the association between inherited metabolic disorders and NT measurements was by De Biasio et al. in 2006. They observed 13 fetuses afflicted with Gaucher disease, glycogenosis type II, mucopolysaccharidosis type I, Krabbe disease, metachromatic leukodystrophy, mucopolysaccharidosis type II, NPD-A, Pelizaeus–Merzbacher disease, and sialidosis, and concluded that an increased NT was found only in one fetus with trisomy 21 but without mucopolysaccharidosis type II. Thus, the NT in early pregnancy might be normal even in fetuses afflicted with conditions known to be associated with non-immune hydrops fetalis (33).

When thin septae are present inside the cervix along with fluid accumulation, differential diagnosis includes an enlarged NT and cystic hygroma. Cystic hygroma or hygroma colli involves the abnormal accumulation of fluid in the region of the fetal neck (34); its diagnosis via ultrasonography is based on a bilateral, mostly symmetric, cystic structure present in the occipitocervical region, with the lesion either septated by internal trabeculae or non-septated (35). Our ultrasonographic findings revealed the presence of fluid accumulation at the level of the fetal neck that was 6 mm in length with thin septae and no evidence of lateral accumulation. In such cases, it is disputed whether the presence of septae alone can differentiate between increased NT and cystic hygroma (36). Other studies found that cystic hygroma is more likely to be associated with genetic anomalies than increased NT, although both conditions require genetic tests for diagnosis (37).

In our case, the cystic hygroma was the main finding that prompted the prenatal diagnosis of NPD-C2. This was of particular importance considering the mother’s first-born child was unaffected. Determining the carrier status of both parents made possible the diagnosis of the disease in the subsequent pregnancy despite the fetus’ normal appearance on ultrasonography.

Regarding future pregnancies, the family was informed of that there was a 25% chance of transmitting the genetic anomaly to a subsequent fetus, and that preimplantational diagnosis is the only option to avoid another afflicted child. The couple refused *in vitro* fertilization and preimplantational diagnosis for personal and social reasons.

## Concluding Remarks

Cystic hygroma in the first trimester was associated with fetal chromosomal or structural anomalies; however, there have been no published reports on the association of NT/CH with NPD-C to date. We describe a fetus in which NPD-C2 was diagnosed in the first trimester based solely on an abnormal NT appearance, which allowed the screening and detection of this disease during the patient’s subsequent pregnancy despite a normal NT.

## Availability of Data and Materials

All data generated or analyzed during this study are included in this published article.

## Ethics Statement

Written consent was obtained from the patient. The patient consented to the publication of medical data (including figures from diagnostic imaging results and from histological examination results).

## Author Contributions

All the authors materially participated in this work; have read and approved the final manuscript. LP and FN carried out the clinical genetic examination of the patient; contributed to the clinical description and molecular genetic description; and conceived, designed, and evaluated the molecular genetic investigation. R-MS and MM co-wrote the manuscript and revised it critically for important intellectual content and for appropriate language.

## Conflict of Interest Statement

The authors declare that the research was conducted in the absence of any commercial or financial relationships that could be construed as a potential conflict of interest.

## Abbreviations

NPD-A, Niemann–Pick disease type A; NPD-B, Niemann–Pick disease type B; NPD-C, Niemann–Pick disease type C; NPD-C2, Niemann–Pick type C2; NT, nuchal translucency.
